# Synergistic effect of vancomycin combined with cefotaxime, imipenem, or meropenem against Staphylococcus aureus with reduced susceptibility to vancomycin

**DOI:** 10.3906/sag-1910-166

**Published:** 2021-08-30

**Authors:** Arpasiri SRİSRATTAKARN, Chonthicha CHAİYAPOKE, Sirikarn BOONCHAROEN, Sujintana WONGTHONG, Aroonwadee CHANAWONG, Patcharaporn TİPPAYAWAT, Ratree TAVİCHAKORNTRAKOOL, Aroonlug LULİTANOND

**Affiliations:** 1 Centre for Research and Development of Medical Diagnostic Laboratories, Faculty of Associated Medical Sciences, Khon Kaen University, Khon Kaen Thailand; 2 Department of Clinical Microbiology, Faculty of Associated Medical Sciences, Khon Kaen University, Khon Kaen Thailand; 3 Faculty of Medical Technology, Nakhon Ratchasima College, Nakhon Ratchasima Thailand

**Keywords:** β-Lactams methicillin-resistant* Staphylococcus aureus*, synergy, vancomycin, vancomycin resistance

## Abstract

**Background/aim:**

We investigated the synergistic effect between vancomycin and β-lactams against vancomycin-susceptible (VSSA) and nonsusceptible MRSA isolates [heterogeneous vancomycin-intermediate *S. aureus* (hVISA) and VISA].

**Materials and methods:**

A total of 29 MRSA, including 6 VISA, 14 hVISA, and 9 VSSA isolates, were subjected to a microbroth dilution-minimum inhibitory concentration (MIC) checkerboard using vancomycin combined with cefotaxime, imipenem, or meropenem. To confirm synergistic activity, the representative strains of VISA, hVISA, and VSSA were then selected for the time-kill curve method.

**Results:**

The combination of vancomycin with imipenem, meropenem, or cefotaxime exhibited synergistic effects against 17 (2 VISA, 9 hVISA, and 6 VSSA), 14 (3 VISA, 9 hVISA and 2 VSSA), and 5 (3 VISA and 2 hVISA) isolates, respectively. Additive and indifferent effects were found in the remaining isolates, but no antagonistic effect was observed. Using time-kill assay, the vancomycin combined with either imipenem or cefotaxime demonstrated synergism against both VISA and hVISA isolates, while the synergistic effect with meropenem was obtained only in the VISA isolates.

**Conclusion:**

This study demonstrated in vitro enhanced antibacterial activity of vancomycin plus β-lactams against clinical hVISA or VISA isolates. These combinations may be an alternative treatment for MRSA infections in clinical practice.

## 1. Introduction

*Staphylococcus aureus* is an important pathogenic bacterium that plays a significant role in human diseases, especially the strain that resists methicillin, called methicillin-resistant *S. aureus* (MRSA). Vancomycin, a glycopeptide antibiotic discovered in 1952, has activity against a wide range of gram-positive bacteria [1].It is often a drug of choice for the treatment of serious infections caused by MRSA. However, clinical MRSA isolates with reduced susceptibility to vancomycin, heterogeneous vancomycin-intermediate *S. aureus* (hVISA), and vancomycin-intermediate *S. aureus* (VISA) have emerged, resulting in poor clinical outcomes [2,3].Vancomycin monotherapy is associated with treatment failure and higher rates of hospitalization and mortality [4]. A combination of antimicrobial agents has therapeutic benefits and leads to rapid recovery of patients [5].

The concept of combination of vancomycin with β-lactams was mentioned a decade ago [6]. Vancomycin combined with β-lactams showed an additive or synergistic effect against MRSA isolates. The β-lactam drugs enhanced vancomycin surface binding, reduced cell wall thickening, and acted as an inhibitor at different stages of cell wall synthesis [3,7,8]. In addition, the synergistic effect helped to reduce the vancomycin dosage, resulting in lowering the risk of nephrotoxicity [9]. Therefore, clinical use of vancomycin and β-lactam combination as an alternative therapy for MRSA with reduced vancomycin susceptibility may be superior to vancomycin monotherapy. However, reports of this combination against MRSA isolates with reduced susceptibility to vancomycin are limited, and the results remain inconsistent. We, thus, evaluated the combination of three β-lactams, including cefotaxime, meropenem, and imipenem with vancomycin against VISA, hVISA, and vancomycin-susceptible *S. aureus* (VSSA) isolates by using a broth microdilution checkerboard and time-kill assays. The combination therapy may provide an option for combating the critical infection caused by hVISA or VISA.

## 2. Materials and methods

### 2.1. Bacterial strains

A total of 29 clinical* S.* *aureus* (6 VISA, 14 hVISA, and 9 VSSA) isolates collected from individual patients attending the Srinagarind Hospital, Khon Kaen University, Thailand between 2010 and 2016 were included. All isolates were identified using conventional biochemical tests such as tube coagulase, phenol red mannitol, and DNase tests, and *mecA* gene was detected using a PCR method [10]. The hVISA phenotype was determined via a population analysis profile with area under the curve (PAP-AUC) [2].

### 2.2. Antimicrobial agents

All antimicrobials used in this study were purchased from commercial sources: cefotaxime (CTX) and vancomycin (VAN) from Sigma-Aldrich (St Louis, USA), imipenem (IPM) from MSD (Whitehouse Station, NJ, USA), and meropenem (MEM) from Siam Bheasach (Bangkok, Thailand).

### 2.3. Population analysis profile with an area under the curve ratio (PAP-AUC ratio)

PAP of hVISA phenotype confirmation used in this study was described in a previous study [11]. Briefly, an overnight bacterial broth culture with turbidity of McFarland standard no. 0.5 was serially 10-fold diluted from 10^0^-10^-6^. An aliquot of 100 µL of each dilution was spread on brain heart infusion agar (BHIA) (Oxoid, Basingstoke, UK) containing various vancomycin concentrations of 0, 0.5, 1, 2, 3, 4, 5, 6, 7, and 8 µg/mL. After incubation at 37 °C for 48 h, bacterial colonies were counted and further converted to a colony-forming unit (CFU). The log10 numbers of CFU/mL were plotted against the vancomycin concentrations using Graph Pad Prism software version 5.0.1 (GraphPad Software Inc., San Diego, USA). The area under the curve (AUC) of each isolate was calculated according to the ratio of the AUC of the test strain and that of the reference hVISA strain (Mu3). PAP-AUC ratio criteria for the determination of VSSA, hVISA, and VISA strains are as described previously [11]: <0.90 = VSSA, 0.90–1.30 = hVISA, and >1.30 = VISA. *S. aureus* ATCC700699 (Mu50, VISA), ATCC700698 (Mu3, hVISA), and ATCC29213 (VSSA) were used as positive control strains of homogeneous, heterogeneous vancomycin resistance and negative control strains, respectively.

### 2.4. Susceptibility testing

The minimum inhibitory concentrations (MICs) and synergistic effect of vancomycin and β-lactam antimicrobials were tested in duplicate by using a microdilution checkerboard technique, which was performed in a 96-well microtiter plates with Mueller-Hinton broth (Oxoid). The susceptibility testing using a broth microdilution method was performed and interpreted according to the CLSI guidelines (MIC breakpoint: susceptible, ≤2 μg/mL; intermediate, 4–8 μg/mL; and resistant, ≥16 μg/mL) [12,13]. The test concentrations of each β-lactam ranged from 0.125 to 64 µg/mL, and those of vancomycin were 0.125, 0.25, 0.5, 1, 2, 3, and 4 µg/mL. The final bacterial inoculum was approximately 10^5 ^CFU/mL. The 96-well plates were incubated at 37 °C for 24 h [14–16], and the first clear well in each row and column containing both antimicrobials was read and calculated as the fractional inhibitory concentration (FIC) index. The FIC index is the FIC of drug A (the MIC of the antimicrobial A in the combination divided by the MIC of the antimicrobial A alone) plus FIC of drug B (the MIC of the antimicrobial B in the combination divided by the MIC of the antimicrobial B alone). The FIC index values of <0.5, 0.5–1.0, >1–4.0, and >4.0 were defined as synergy, additive, indifference, and antagonism, respectively [17]. Growth and sterility controls were tested in each test panel. In addition, *S. aureus* ATCC29213 strain was used as a control strain.

### 2.5. Time-kill assay

The synergy of VAN plus IPM, CTX, or MEM was performed by using an inoculum of ~10^6^ CFU/mL in MHB at sub-MICs (one-half of MIC) of the antimicrobials. Tubes without antimicrobial were used for growth control. Bacterial counts were taken at 0, 2, 4, 8, and 24 h. Synergy between VAN and each β-lactam was deﬁned as a ˃2 log10 CFU/mL decrease of the combination over the most active single agent after 24 h and ≥1 log10 CFU/mL reduction from baseline [7].

## 3. Results

**Figure 1:**
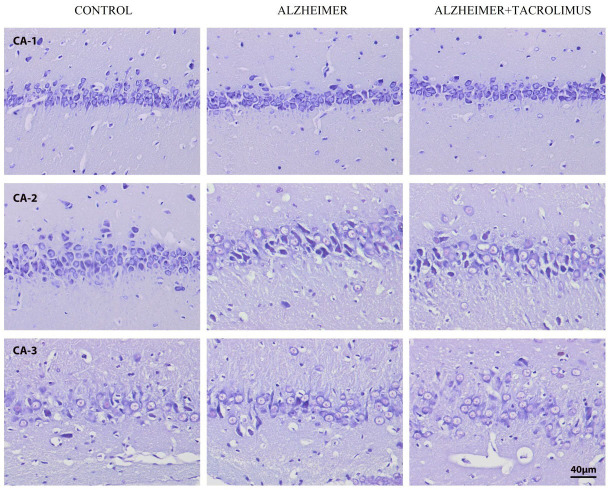
Comparison of the mean MIC values of vancomycin (VAN) alone and in combination with cefotaxime, CTX; meropenem, MEM; imipenem, IPM against 6 vancomycin-intermediate S. aureus (VISA), 14 heterogeneous VISA (hVISA) and 9 vancomycin-susceptible S. aureus (VSSA) isolates.

The ranges of VAN MIC against 6 VISA, 14 hVISA, and 9 VSSA isolates were 3– > 4, 1–2, and 1–2 μg/mL, respectively. The MIC ranges for CTX, IPM, and MEM were 16– > 64, 4– > 64, and 4– > 64 μg/mL; 0.125–2, 0.125–64, and 0.125–64 μg/mL; and 0.25–16, 2– > 64, and 0.25–64 μg/mL, respectively. The MICs of VAN in combination with CTX, MEM, or IPM showed 1–4, 2–5, and 2–6 dilutions less than those of the VAN alone. Likewise, when CTX, MEM, or IPM was combined with VAN, the MICs of each agent also reduced 2–9, 2–8, and 1–9 dilutions to those of each agent alone, respectively (Tables 1 and 2). The mean MICs of VAN when combined with IPM for the VISA, hVISA, and VSSA isolates showed 91.8%, 82%, and 76.2% reduction from those of the VAN alone, respectively. The VAN plus either CTX or MEM also had similar activities to decrease the MICs of VAN from those using the VAN alone for VSSA group (36.1% and 63.9% decreased, respectively) (Figure 1).

**Table 1:** Fractional inhibitory concentration indexes of vancomycin plus cefotaxime, meropenem, or imipenem combination against 29 Staphylococcus aureus using a checkerboard technique.

Strains	MIC (µg/mL)			FIC index	MIC (µg/mL)		FIC index	MIC (µg/mL)		FIC index
	VAN	CTX	VAN + CTX		MEM	VAN + MEM		IPM	VAN + IPM	
VISA (6)	3– > 4	16– > 64	VAN = 0.25–2CTX = 0.5–64	0.33–0.67(Sy = 3, Ad = 3)	0.25–16	VAN = 0.25–2MEM = 0.125–2	0.38–0.67 (Sy = 3, Ad = 3)	0.125–2	VAN = 0.125–1IPM = 0.125	0.23–1.04 (Sy = 2, In = 4)
hVISA(14)	1–2	4– > 64	VAN = 0.25–1CTX = 0.25–64	0.19–0.75(Sy = 2, Ad = 12)	2– > 64	VAN = 0.25–1MEM = 0.25–8	0.19–0.75 (Sy = 9, Ad = 5)	0.125–64	VAN = 0.125–0.5IPM = 0.125–2	0.06–1.13 (Sy = 9, Ad = 2, In = 3)
VSSA(9)	1–2	4– > 64	VAN = 0.5–1CTX = 0.25–32	0.50–1.01(Ad = 9)	0.25–64	VAN = 0.25–1MEM = 0.125–32	0.38–1.00 (Sy = 2, Ad = 7)	0.125–64	VAN = 0.125–0.5IPM = 0.125–0.5	0.13–1.13 (Sy = 6, Ad = 2, In = 1)
Total (29)				Sy = 5, Ad = 24			Sy = 14, Ad = 15			Sy = 17, Ad = 4, In = 8

**Table 2:** Fractional inhibitory concentration indexes of vancomycin plus cefotaxime, meropenem, or imipenem combinations against each Staphylococcus aureus isolates using a checkerboard technique.

Strains	MIC (µg/mL)			FIC index	MIC (µg/mL)		FIC index	MIC (µg/mL)		FIC index
	VAN	CTX	VAN + CTX		MEM	VAN + MEM		IPM	VAN + IPM	
VI 123	3	16	0.25 + 4	0.33 (Sy)	0.25	0.5 + 0.125	0.67 (Ad)	0.125	0.125 + 0.125	1.04 (In)
VI 127	4	>64	0.5 + 64	0.63 (Ad)	0.5	0.25 + 0.25	0.56 (Ad)	0.125	0.125 + 0.125	1.03 (In)
VI 152	3	>64	2 + 0.5	0.67 (Ad)	16	1 + 1	0.39 (Sy)	2	0.5 + 0.125	0.23 (Sy)
VI 214	3	>64	0.5 + 32	0.42 (Sy)	1	1 + 0.25	0.58 (Ad)	0.125	0.125 + 0.125	1.04 (In)
VI 7	3	64	1 + 4	0.39 (Sy)	4	1 + 0.25	0.39 (Sy)	0.125	0.125 + 0.125	1.04 (In)
VI 17	>4	32	2 + 8	0.50 (Ad)	16	2 + 2	0.38 (Sy)	1	1 + 0.125	0.25 (Sy)
hVI 134	1	>64	0.25 + 0.5	0.25 (Sy)	64	0.25 + 8	0.38 (Sy)	16	0.5 + 0.125	0.51 (Ad)
hVI 250	1	>64	0.5 + 32	0.75 (Ad)	64	0.25 + 4	0.31 (Sy)	64	0.25 + 2	0.28 (Sy)
hVI 261	2	>64	1 + 4	0.53 (Ad)	32	0.5 + 1	0.28 (Sy)	32	0.5 + 0.125	0.25 (Sy)
hVI 276	2	>64	1 + 4	0.53 (Ad)	32	0.5 + 1	0.28 (Sy)	32	0.125 + 0.125	0.06 (Sy)
hVI 280	2	64	0.25 + 4	0.19 (Sy)	2	0.25 + 1	0.63 (Ad)	0.125	0.125 + 0.125	1.06 (In)
hVI 297	1	4	0.25 + 2	0.75 (Ad)	16	0.25 + 1	0.31 (Sy)	0.125	0.125 + 0.125	1.13 (In)
hVI 300	2	>64	1 + 0.25	0.50 (Ad)	>64	0.25 + 8	0.19 (Sy)	1	0.25 + 0.125	0.25 (Sy)
hVI 302	1	64	0.25 + 16	0.50 (Ad)	2	0.25 + 1	0.75 (Ad)	0.125	0.125 + 0.125	1.13 (In)
hVI 17	2	>64	1 + 2	0.52 (Ad)	64	1 + 0.25	0.50 (Ad)	1	0.5 + 0.25	0.50 (Ad)
hVI 1	1	>64	0.5 + 64	1.00 (Ad)	8	0.25 + 2	0.50 (Ad)	64	0.25 + 2	0.28 (Sy)
hVI 7	1	>64	0.5 + 8	0.56 (Ad)	4	0.25 + 1	0.50 (Ad)	16	0.25 + 1	0.31 (Sy)
hVI 8	1	>64	0.5 + 8	0.56 (Ad)	16	0.25 + 2	0.38 (Sy)	4	0.25 + 0.5	0.38 (Sy)
hVI 9	2	>64	1 + 4	0.53 (Ad)	32	0.25 + 4	0.25 (Sy)	16	0.25 + 0.125	0.13 (Sy)
hVI 13	2	>64	1 + 2	0.52 (Ad)	16	0.50 + 1	0.31 (Sy)	8	0.25 + 0.125	0.14 (Sy)
VS 66	1	>64	0.5 + 32	0.75 (Ad)	32	0.5 + 4	0.63 (Ad)	32	0.5 + 0.5	0.52 (Ad)
VS 67	1	>64	1 + 1	1.01 (Ad)	8	0.5 + 1	0.63 (Ad)	32	0.5 + 0.125	0.50 (Ad)
VS 68	1	>64	1 + 0.25	1.00 (Ad)	16	0.25 + 8	0.75 (Ad)	8	0.25 + 0.5	0.31 (Sy)
VS 70	1	>64	0.5 + 16	0.63 (Ad)	16	0.25 + 2	0.38 (Sy)	4	0.25 + 0.125	0.28 (Sy)
VS 71	2	>64	1 + 0.5	0.50 (Ad)	16	0.25 + 4	0.38 (Sy)	2	0.125 + 0.125	0.13 (Sy)
VS 72	1	>64	1 + 0.25	1.00 (Ad)	64	0.25 + 32	0.75 (Ad)	64	0.125 + 0.125	0.13 (Sy)
VS 8	1	4	0.5 + 0.5	0.63 (Ad)	0.25	0.5 + 0.125	1.00 (Ad)	0.125	0.125 + 0.125	1.13 (In)
VS 12	2	>64	1 + 8	0.56 (Ad)	64	1 + 0.5	0.51 (Ad)	64	0.5 + 0.125	0.25 (Sy)
VS 31	1	>64	0.5 + 32	0.75 (Ad)	16	0.5 + 2	0.63 (Ad)	16	0.25 + 0.25	0.27 (Sy)

* FIC index: <0.5: synergy (Sy); 0.5–1.0: additive (Ad); > 1–4.0: indifference (In); >4.0: antagonism (An) [17].

The VAN plus IPM showed the highest synergistic effect against 17 of the 29 isolates (58.6%; 2 VISA, 9 hVISA, and 6 VSSA isolates). Similarly, the VAN plus MEM had synergistic effects against 14 isolates (48.3%; 3 VISA, 9 hVISA, and 2 VSSA isolates). In contrast, the VAN plus CTX gave synergistic effect against 5 isolates only (17.2%; 3 VISA and 2 hVISA), whereas the additive results were found in most isolates (Table 1). However, a synergistic effect of VAN plus either CTX or MEM was found against a VISA isolate with high level of VAN MIC (>4 μg/mL) (Table 2). In addition, no antagonistic result was observed in any isolates. 

Among the 3 couples of antimicrobials, the VAN plus IPM had higher inhibitory effectiveness than the other two pairs (mean FIC indexes was 0.23 in the synergistic activity group). The synergistic effect (FIC indexes of ≤0.5) was found in most isolates with high MICs (≥16 μg/mL) of CTX (100%), MEM (93%), and IPM (53%) (Table 2).

**Figure 2:**
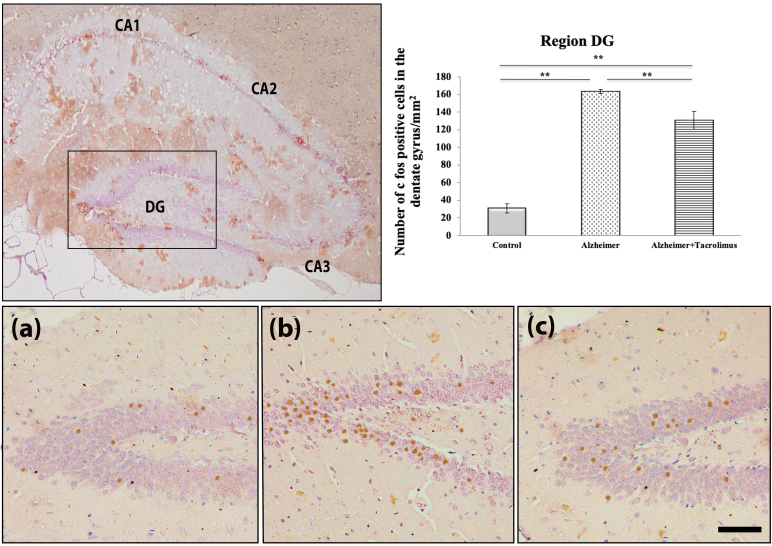
Cumulative percentages (%) of synergistic activities of the vancomycin (VAN) and β-lactam (imipenem, IPM; meropenem, MEM; cefotaxime, CTX) combinations affected by various concentrations of β-lactams (solid lines) and vancomycin (dashed lines) against 29 test isolates.

Notably, the combination of VAN with 0.125 µg/mL of IPM showed indifference and synergistic effects against most of the isolates (8 and 11 isolates respectively), the cumulative percentage of synergistic effect between VAN and IPM rising to 82.4% when 0.5 mg/L of IPM was used, whereas those of the VAN plus MEM and VAN plus CTX were 42.9% and 20% when 1 µg/mL of MEM or CTX were used respectively (Figure 2).

**Figure 3:**
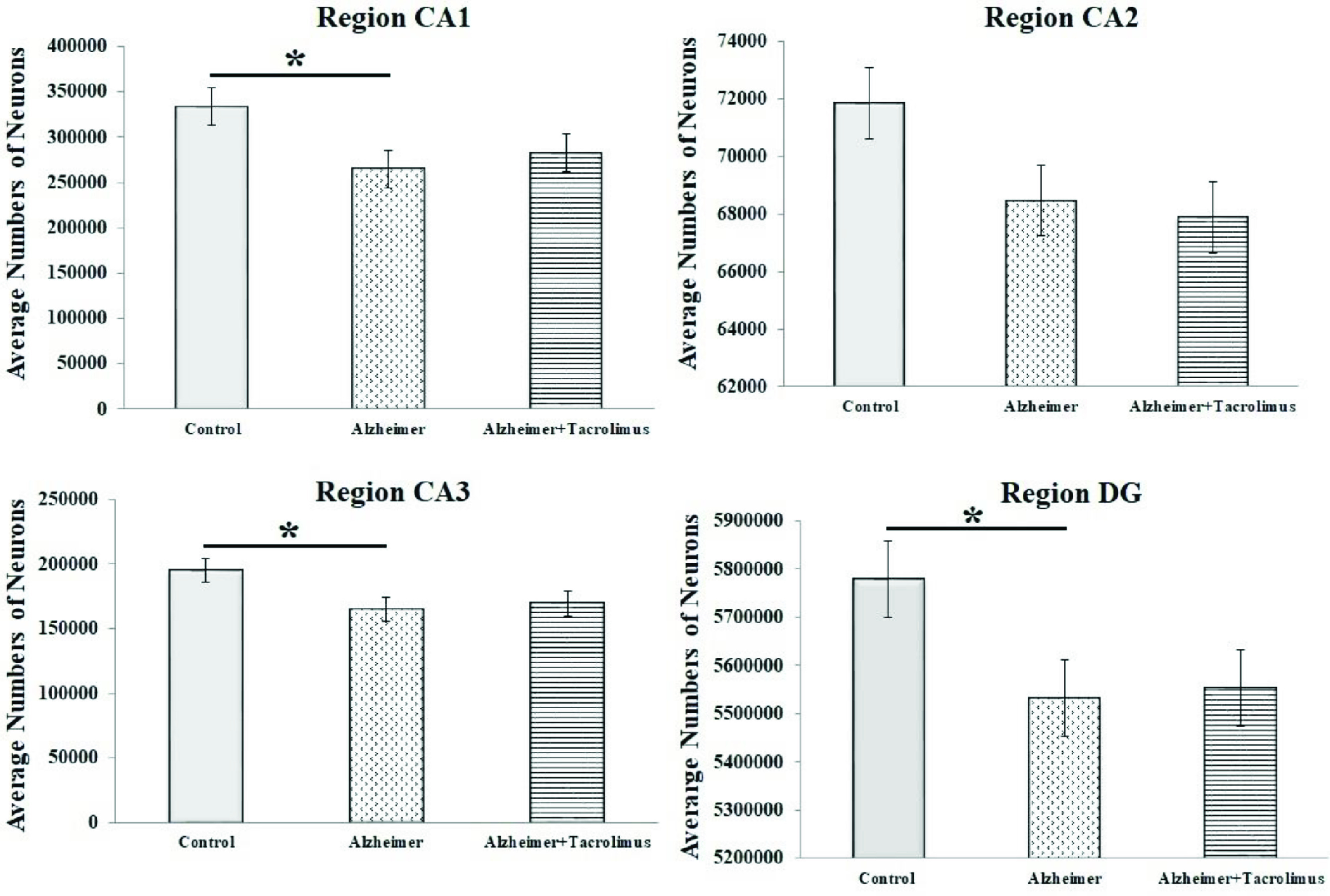
Time-kill curves of each antimicrobial (solid lines) and their combinations (dashed lines) against VISA (a), hVISA (b), and VSSA (c) strains.Growth controls (black lines), vancomycin (blue diamonds), imipenem (red circles), cefotaxime (green triangles), and meropenem (yellow squares).

To confirm the synergistic effects determined using the checkerboard method, the representative strains of VISA, hVISA, and VSSA (isolate no. VI 152, hVI 300, and VS 71, respectively) were selected for the time-kill assay. The mean 24-h reductions of bacterial counts for VAN plus IPM, VAN plus MEM, and VAN plus CTX were 4, 3.67, and 3 log10 CFU/mL, respectively. The VAN plus IPM or CTX showed synergy against VISA (Figure 3a) and hVISA strains (Figure 3b) within 24 h of incubation, whereas synergism by the VAN plus MEM was observed in the VISA strain only. The time-kill assay of VAN plus β-lactams showed no synergistic effect for the VSSA strain (Figure 3c). 

## 4. Discussion

Carbapenems and the 3^rd^ generation cephalosporins have an extremely broad spectrum of antimicrobial activity against both gram-positive and gram-negative bacteria. Therefore, we tested the activity of IPM, MEM, and CTX combined with VAN against MRSA isolates. 

The increasing use of VAN has caused a selective pressure, leading to the occurrence of vancomycin-resistant strains. This resulted in the therapeutic failure, morbidity, and even death [2]. Due to limited options of therapeutic drugs, several studies have focused on the combination of antimicrobials as an alternative treatment. The appropriate antimicrobial treatments provided effective therapy, reducing antimicrobial doses and adverse effects and decreased both cost and length of hospitalization. 

In this study, synergy effect of the combined drugs was found in varying numbers of the vancomycin-susceptible and nonsusceptible MRSA isolates. Although the combinations of these β-lactams and VAN were not synergistic against all isolates, no antagonistic effect was found. These results suggested that the additive and indifferent effects may have been the consequences of the method’s limitation since the antimicrobials were applied in various concentrations. Therefore, the real effect may be synergistic rather than additive effects [18]. However, the checkerboard technique was mostly used as a reference method for determining synergy of drugs [16]. Our results supported that the FIC indexes of the β-lactam-VAN combination inversely correlated with the MICs of the β-lactam alone [6]. Most cases of synergistic effects (FIC indexes of <0.5) occurred in the strains that had high MIC for CTX, MEM, and IPM. Among the three β-lactams tested, IPM was considered to be the best agent to combine with VAN, frequently showing a synergistic effect, particularly against hVISA strains. In addition, the synergistic effect of VAN plus IPM can be enhanced at a lower IPM concentration (0.125 μg/mL), compared with MEM (1 μg/mL) and CTX (0.5 μg/mL). The concentrations found to have a synergistic effect are clinically accessible and revealed within the range of MIC breakpoint of CLSI [13]. The vancomycin plus β-lactams demonstrated an enhanced antibacterial effect at susceptible breakpoint concentrations. Both β-lactams and VAN have activity against bacteria by preventing the biosynthesis of the bacterial cell wall. The activity of β-lactam targets at the transpeptidase enzymes, which manage the crosslink of peptidoglycan in the bacterial cell wall. In addition, the β-lactam also alters the bacterial cell surface, which helps to access the specific target for the binding of VAN [19]. On the other hand, the target site of VAN is pentapeptide side chain, leading to inhibition of transglycosylation and transpeptidation. Moreover, VAN also alters the permeability of the cell membrane and selectively inhibits ribonucleic acid synthesis [20].These activities promote the synergistic effect of their combinations.

In this study, the synergistic activity of antimicrobial combinations was confirmed by the time-kill assay. Our data supported the results of the checkerboard method that VAN combined with β-lactams demonstrates synergistic activity against staphylococcal isolates with reduced susceptibility to VAN. Interestingly, the mean 24-h of bacterial reduction for VAN plus IPM was the highest compared with the other combinations.

IPM is a potent β-lactam antimicrobial that has a postantibiotic effect (PAE) against gram-positive bacteria and resists the hydrolysis by most β-lactamases [21,22]. Although the MRSA strains are not susceptible to this agent, several studies have reported the efficacy of IPM when used in combination with other antimicrobials, including cephalosporins and vancomycin [14,15,18,23,24], thus corresponding with this study. Therefore, the use of unconventional combinations of drugs may be an alternative for the management of MRSA isolates with reduced susceptibility to VAN. 

In the present study, some limitations should be noted; a few strains of VISA have been observed due to the prevalence of clinical VISA in our area; thus, larger samples should be evaluated in further studies. In addition, these combinations should be investigated in clinical or in vivo conditions to support the recommendation of β-lactam combination therapy in routine clinical use. However, few studies have investigated animal models for the combinations of VAN with β-lactams, including nafcillin, imipenem, or ceftobiprole, and they have found evidence of synergy [6,25,26]. In addition, clinical studies revealed an increasing rate of microbiological eradication when using the combination of VAN with piperacillin-tazobactam or β-lactams in therapeutic groups [27–29].

In conclusion, this is an in vitro study that used checkerboard and time-kill assays to determine the activity of VAN and β-lactam combinations, which demonstrated the enhanced antibacterial activity against clinical hVISA or VISA isolates, suggesting that it may be an alternative for use in clinical therapy.

## Informed consent

This study was conducted in accordance with the Declaration of Helsinki and was approved by the Ethics Committee of Khon Kaen University (project number HE552272).
